# Application of preoperative advanced diffusion magnetic resonance imaging in evaluating the postoperative recurrence of lower grade gliomas

**DOI:** 10.1186/s40644-024-00782-9

**Published:** 2024-10-09

**Authors:** Luyue Gao, Yuanhao Li, Hongquan Zhu, Yufei Liu, Shihui Li, Li Li, Jiaxuan Zhang, Nanxi Shen, Wenzhen Zhu

**Affiliations:** 1 Department of Radiology, Tongji Hospital, Tongji Medical College, Huazhong University of Science and Technology, 1095 Jiefang Avenue, Wuhan, 430030 PR China; 2 Department of Radiology, Qianjiang Central Hospital, 22 Zhanghua Middle Road, Qianjiang, 433100 PR China

**Keywords:** Diffusion magnetic resonance imaging, Glioma, Recurrence

## Abstract

**Background:**

Recurrence of lower grade glioma (LrGG) appeared to be unavoidable despite considerable research performed in last decades. Thus, we evaluated the postoperative recurrence within two years after the surgery in patients with LrGG by preoperative advanced diffusion magnetic resonance imaging (dMRI).

**Materials and methods:**

48 patients with lower-grade gliomas (23 recurrence, 25 nonrecurrence) were recruited into this study. Different models of dMRI were reconstructed, including apparent fiber density (AFD), white matter tract integrity (WMTI), diffusion tensor imaging (DTI), diffusion kurtosis imaging (DKI), neurite orientation dispersion and density imaging (NODDI), Bingham NODDI and standard model imaging (SMI). Orthogonal Partial Least Squares-Discriminant Analysis (OPLS-DA) was used to construct a multiparametric prediction model for the diagnosis of postoperative recurrence.

**Results:**

The parameters derived from each dMRI model, including AFD, axon water fraction (AWF), mean diffusivity (MD), mean kurtosis (MK), fractional anisotropy (FA), intracellular volume fraction (ICVF), extra-axonal perpendicular diffusivity (De^⊥^), extra-axonal parallel diffusivity (De^∥^) and free water fraction (fw), showed significant differences between nonrecurrence group and recurrence group. The extra-axonal perpendicular diffusivity (De^⊥^) had the highest area under curve (AUC = 0.885), which was significantly higher than others. The variable importance for the projection (VIP) value of De^⊥^ was also the highest. The AUC value of the multiparametric prediction model merging AFD, WMTI, DTI, DKI, NODDI, Bingham NODDI and SMI was up to 0.96.

**Conclusion:**

Preoperative advanced dMRI showed great efficacy in evaluating postoperative recurrence of LrGG and De^⊥^ of SMI might be a valuable marker.

## Introduction

Lower grade gliomas (LrGG) refer to WHO grade II and III gliomas [1]. Compared with glioblastoma (GBM), the prognosis of LrGG is relatively better. However, the probability of postoperative malignant transformation and recurrence is still high due to its invasive growth characteristics [2]. Surgery is still the main treatment for recurrent gliomas, but there still exists a lack of high-level evidence-based medicine for the survival benefits of patients. Therefore, the prediction of postoperative recurrence has a certain significance in clinic, which may provide convenience for neurosurgeons to evaluate the prognosis of patients and to take targeted precautions, such as appropriate expansion of the resection scope and usage of anti-angiogenic drugs [3].

At present, there is no comprehensive method to predict the recurrence of glioma after operation, but previous researches have shown that the main prognostic risk factors of LrGG included age [4], tumor position [4, 5], size [6] and concurrent chemoradiotherapy [7, 8]. Meanwhile, many molecular phenotypes are also important factors leading to poor prognosis of LrGG, such as IDH1 [9], MGMT [10], CDKN2A/B [11–13]. Although pathology through surgery or biopsy is the gold standard for obtaining these key molecular phenotypes, this method is not suitable for all types of gliomas because of its invasiveness and tumor heterogeneity. The existing studies focused exclusively on high-grade glioma. Therefore, an accurate and relatively non-invasive in vivo imaging technique that can predict lower-grade glioma recurrence is necessary for optimal management that avoids the risk of reoperation or rebiopsy and related complications.

Diffusion magnetic resonance imaging (dMRI) is a quantitative imaging technique that can detect the microscopic dispersion characteristics of water molecules in tissue. Diffusion tensor imaging (DTI) and diffusion-kurtosis imaging (DKI) metrics have been used for differentiating tumor recurrence from treatment effects [14, 15]. However, the diagnostic performance of DTI for determining treatment response has been shown to be inconsistent in various studies [16, 17], and some have found that DKI metrics do not appear to provide additional prognostic value compared to other imaging techniques [18, 19]. Recently, novel diffusion MRI techniques, such as the neurite orientation dispersion and density imaging (NODDI) and standard model imaging (SMI) have emerged as powerful tools to evaluate brain microstructure in vivo, as they can provide new insights into the complexity and inhomogeneity of brain microstructure [20, 21]. NODDI metrics have shown promising results in grading of gliomas, assessment of IDH mutation status and monitoring treatment response [22, 23]. However, the value of SMI and NODDI for predicting lower-grade glioma recurrence is unclear. In addition, White Matter Tract Integrity (WMTI) metric, such as axonal water fraction (AWF) is defined as the ratio of intra-axonal water to the sum of intra-axonal water and extra-axonal water which provide more specific insights about tumor physiology [24, 25]. Meanwhile, apparent fiber density (AFD), a dMRI framework to provide a relative measure of the intra-axonal volume of each tract within a voxel, has been used in numerous applications including gliomas. However, to our knowledge, there have no reports on comparison the multiple diffusion models for predicting postoperative recurrence of LrGG via Orthogonal Partial Least Squares-Discriminant Analysis (OPLS-DA).

Therefore, the purpose of this study is to explore the value of preoperative advanced dMRI (AFD, WMTI, DTI, DKI, NODDI, and SMI) in evaluating the postoperative recurrence of LrGG.

## Methods

### Patient Population

This retrospective study was approved by local ethics committee and written informed consent was waived from all subjects. Patients were included in the study if they met the following inclusion criteria: (a) pathologically confirmed primary LrGG; (b) preoperative dMRI acquisition was performed; (c) initial lesion has received grossly total resection; (d) MRI imaging of patients two years after surgery was available. The following were exclusion criteria: (a) lack of routine MRI imaging; (b) initial lesion hasn’t been total resected; (c) MRI imaging after surgery was not available; (d) head trauma or other neurological diseases existed. Any tumor progression could be established radiologically (follow-up imaging), clinically, or histologically. 7 patients were assessed based on imaging alone, refer to Response Assessment in Neuro-Oncology (RANO) criteria, the criteria of recurrence was the new enhancing component within or without surgery area, there is a lesion diameter increase of ≥ 25%, or/and clinical deterioration or new neurological deficits. From November 2013 to January 2020, a total of 169 patients meeting the above criteria were enrolled in this study. 42 cases lack of routine MRI imaging, 3 cases without complete resection and 76 cases without after surgery MRI imaging were excluded. In total, 48 patients were recruited into this study.

### Imaging Data Acquisition

All MR images were performed on the a 3T MR system (Discovery MR750, GE Medical Systems, Milwaukee, WI, United States) with a 32-channel head coil. Routine axial sequences include T1 fluid-attenuated inversion recovery (FLAIR), T2 FLAIR, T2 fast spin echo (FSE) and contrast-enhanced T1 (T1-CE). Before the injection of contrast agents, DKI was performed by spin-echo echo planar imaging (SE-EPI) with 3 b-values (b = 0, 1,250, and 2,500s/mm^2^) and 25 uniformly distributed directions for each nonzero b-value, TR/TE = 6,500/85, NEX = 1, matrix = 128 × 128, slice thickness = 3 mm, spacing = 0 mm, FOV = 240 mm × 240 mm, acquisition time was 5 min 45 s.

### Model Reconstruction

After head motion correction, eddy current correction, bias field correction and brain extraction, several models of dMRI were reconstructed, including apparent fiber density (AFD), axon water fraction (AWF), diffusion tensor imaging (DTI), diffusion kurtosis imaging (DKI), neurite orientation dispersion and density imaging (NODDI), Bingham NODDI and standard model imaging (SMI). SMI model was calculated from https://github.com/NYU-DiffusionMRI/SMI.

The parametric map of DTI was calculated using the data collected by DKI with b = 0 and 1250s/mm^2^. The model was based on Gaussian distribution, and the formula was as follows [26]:

1 $$\:FA=\sqrt{\frac{{\left({\lambda\:}_{1}-{\lambda\:}_{2}\right)}^{2}+{\left({\lambda\:}_{1}-{\lambda\:}_{3}\right)}^{2}+{\left({\lambda\:}_{2}-{\lambda\:}_{3}\right)}^{2}}{2\left({\lambda\:}_{1}^{2}+{\lambda\:}_{2}^{2}+{\lambda\:}_{3}^{2}\right)}}$$

2 $$\:MD= ({\lambda\:}_{1}+{\lambda\:}_{2}+{\lambda\:}_{3})/3$$

While $\:AD={\lambda\:}_{1}$, $\:RD= ({\lambda\:}_{2}+{\lambda\:}_{3})/2$. Derived parameters included axial diffusivity (AD), mean diffusivity (MD), radial diffusivity (RD) and fractional anisotropy (FA).

Based on non-Gaussian distribution, DKI was obtained using following formula:

3 $$\:\frac{S\left(b\right)}{S\left(0\right)}=\text{e}\text{x}\text{p}(-b\cdot\:{D}_{app}+1/6{b}^{2}\cdot\:{D}_{app}^{2}\cdot\:{K}_{app})$$

$\:{D}_{app}$ referred to apparent diffusion coefficient, $\:{K}_{app}$ referred to apparent diffusion kurtosis. Derived parameters included axial kurtosis (AK), mean kurtosis (MK) and radial kurtosis (RK).

NODDI was processed based on Watson and Gaussian distribution, and the formula was as follow:

4 $$\:S=\left(1-{V}_{iso}\right)\left({V}_{ic}{S}_{ic}+\left(1-{V}_{ic}\right){S}_{ec}\right)+{V}_{iso}{S}_{iso}$$

Derived parameters included intracellular volume fraction (ICVF) and orientation dispersion index (ODI). Similarly, Bingham NODDI has been calculated based on the assumption that the way of water molecular motion measured up Bingham distribution, and the derived parameters included Bin-ICVF, Bin-ODI and dispersion anisotropy index (DAI) [27].

Based on non-Gaussian distribution, SMI was obtained using following formula [21]:

5 $$\:S\left(\varvec{B}\right)={s}_{0}{\int\:}_{{\mathbb{S}}^{2}}d\widehat{\varvec{n}}\mathcal{P}\left(\widehat{\varvec{n}}\right)\mathcal{K}(b,\beta\:,\widehat{\varvec{n}}\cdot\:\widehat{\varvec{u}})$$

$\:\mathcal{P}\left(\widehat{\varvec{n}}\right)$ represented orientation distribution function (ODF) and $\:\mathcal{K}\:(b,\:\beta\:,\widehat{\varvec{n}}\cdot\:\widehat{\varvec{u}})$ represented response kernel coded by B-tensor. Derived parameters included axonal diffusivity (Da), extra-axonal parallel diffusivity (De^∥^), extra-axonal perpendicular diffusivity (De^⊥^), fraction (f) and free water fraction (fw).

According to fiber orientation distribution function (fODF), AFD was calculated in Tractflow [28], the derived parameters included AFD-sum, AFD-total and AFD-max. In addition, AWF map was obtained by using white matter tract integrity (WMTI).

### Region of Interest Analysis

Under the premise that researchers were blinded to clinical characteristics, five to eight regions of interest (ROIs) ranging from 80 to 110 pixels were manually placed in the solid parts of tumor parenchyma avoiding hemorrhage, calcification, edema, necrosis and cystic lesions. The diameter of ROIs is 6.0 mm. The solid part of tumor parenchyma was defined as the area of enhancement on T1-CE. For cases with very subtle enhancement, we placed ROIs in high signal intensity area in DWI imaging and T2 FLAIR. We put ROIs for standardizing from the contralateral healthy white matter of the brain, the diameter of ROIs was also 6.0 mm. Then, ROI was copied to each parametric map and the derived parameters value from all ROIs for each patient were recorded for later analysis.

### Statistic analysis

Chi-square tests or R × C columnar tables were used to test categorical variables. According to the normality of continuous variables, t-test was used when data emerged on normal distribution while Mann–Whitney U test was used to analyze data emerging on non-normal distribution. Receiver operating characteristic (ROC) curves were performed to evaluate the diagnostic efficacy of each parameter. And area under the curve (AUC) value was compared by Z test. Orthogonal partial least squares-discriminant analysis (OPLS-DA) was used to construct a multiparametric prediction model with variable importance for the projection (VIP) of each parameter been calculated. All statistical analysis was performed with SPSS (Version 19.0.0, IBM, Armonk, NY, United States), MedCalc (Version 15.8, MedCalc Software, Acacialaan, Ostend, Belgium) and SIMCA (Version 14.1.0.2047, MSK, Umetrics, AB). *P* < 0.05 was considered statistically significant.

## Results

### Clinical characteristics and routine MRI features

A total of 48 LrGG patients were enrolled, including 25 non-recurrence cases and 23 recurrence cases. The differences in lesion margin (*P* = 0.010) and pathological grading (*P* = 0.020) between two groups were statistically significant (Table 1).

**Table 1:** Clinical characteristics and routine MRI features

	Non-recurrence	Recurrence	*P*-value
Number	25	23	N/A
Age^a^	40.17 ± 11.87	43.16 ± 12.53	0.549
Gender			0.971
Male	14	10	
Female	11	13	
Midline Invasion			0.882
Yes	7	6	
No	18	17	
Lesion location			0.571
Frontal Lobe	11	12	
Non-Frontal Lobe	14	11	
Lesion Size			0.154
<6 cm	9	13	
≥ 6 cm	16	10	
**Lesion Margin**			**0.010**
Sharp	19	9	
Blurred	6	14	
Histological Classification			0.309
Astrocytoma	18	16	
Oligodendroglioma	3	6	
Unclear	4	1	
**Pathological Grading**			**0.020**
II	11	17	
III	14	5	
**Therapy after surgery**			0.428
Chemotherapy and radiotherapy	10	16	
Chemotherapy	2	1	
Radiotherapy	7	5	
Not clear	6	3	

^a^Continuous variables were described as means ± standard deviations; N/A, not applicable

### Assessment of Postoperative recurrence of LrGG by Advanced dMRI

**Fig. 1:**
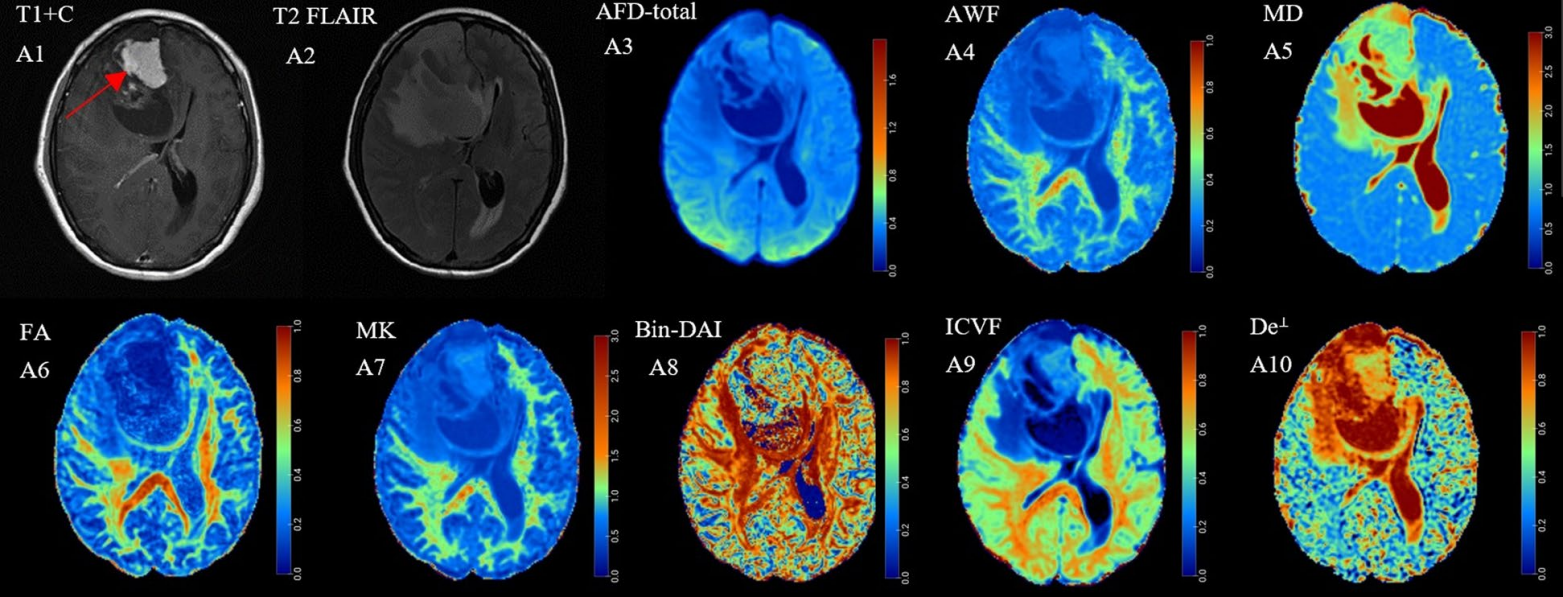
(A1-A10) Correspond to a 48-year-old female patient with astrocytoma (WHO Grade II) who didn’t experience postoperative recurrence within two years after resection. The AFD-total, AWF, FA, MK as well as ICVF maps showed decreased values in the solid part of the tumor (indicated by the red arrow), while MD and De^⊥^ maps showed increased values. No significant difference was observed in the Bin-DAI map

**Fig. 2:**
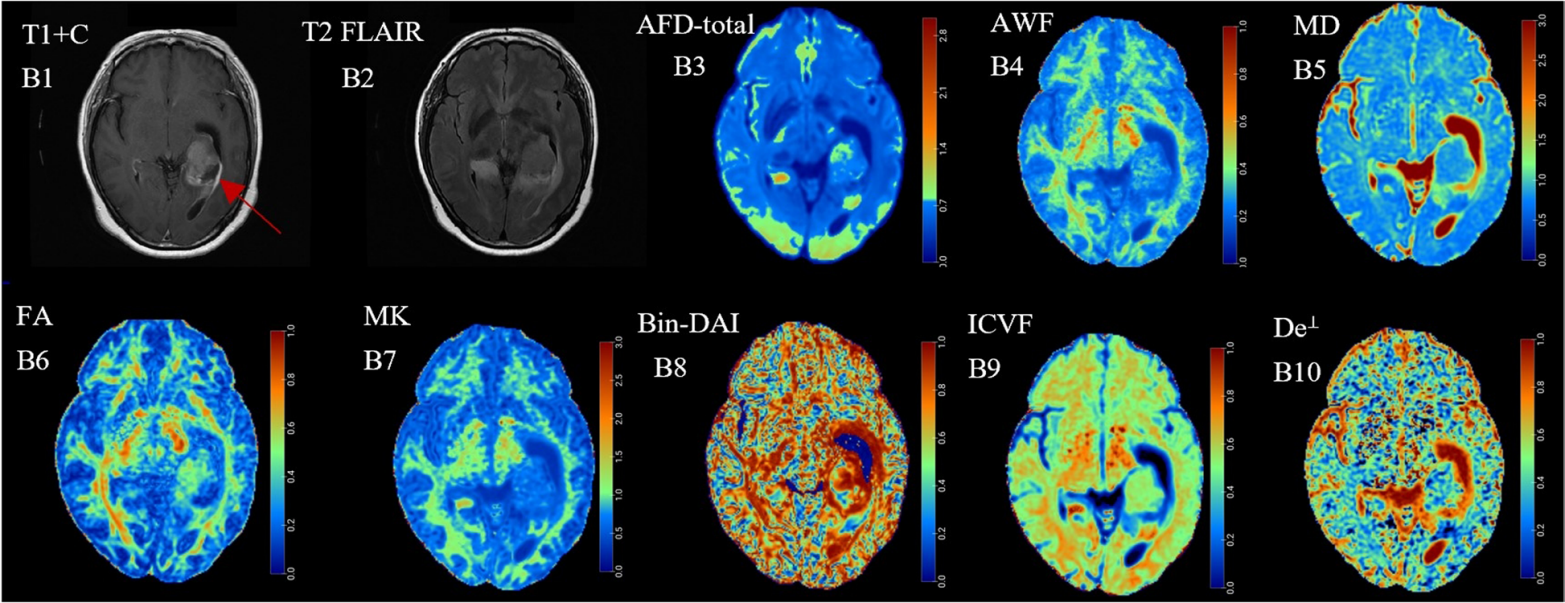
(B1-B10) Correspond to a 51-year-old female patient with astrocytoma (WHO Grade III) who experienced postoperative recurrence within two years after resection. Compared with Fig. 1, the AFD-total, AWF, FA, MK as well as ICVF maps showed increased values in the solid part of the tumor (indicated by the red arrow), while MD and De^⊥^ maps showed decreased values. The difference in Bin-DAI was also not significant

**Fig. 3:**
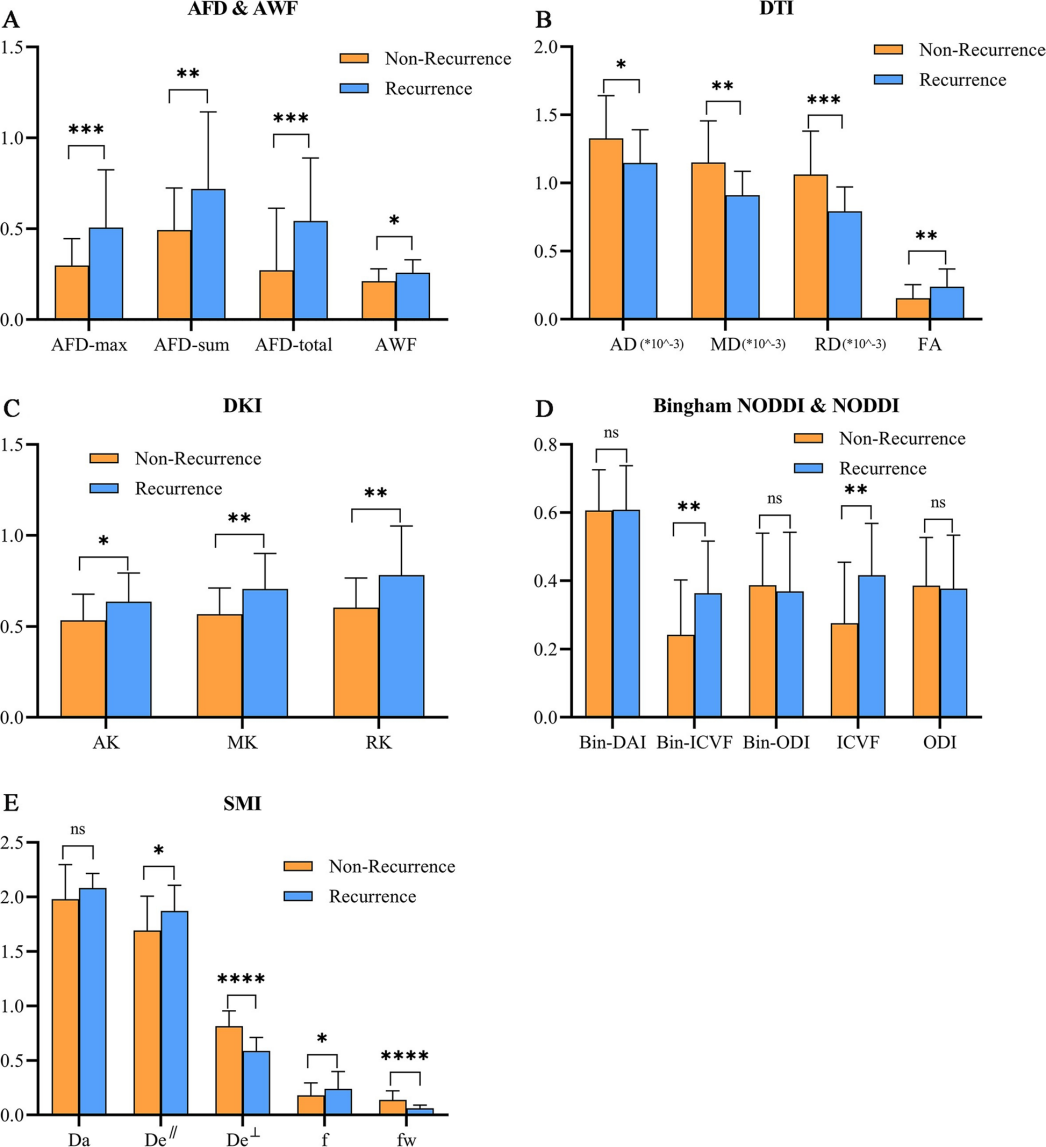
Bar and plots of parameters. Bar represented the mean values while plot extending represented standard deviation. y-axis represents the value of each parameter. *, *P*<0.05; **, *P*<0.01; ***, *P*<0.001; ****, *P*<0.0001; ns, no significance

Figures 1 and 2 showed the preoperative T1-CE, T2 FLAIR and dMRI derived parametric maps of typical recurrence and non-recurrence case. Compared with cases in non-recurrence group, cases in recurrence group showed significantly higher AFD-max, AFD-sum, AFD-total, AWF, FA, AK, MK, RK, Bin-ICVF, ICVF, De^∥^ as well as f values (*P* < 0.05) and lower AD, MD, RD, De^⊥^ as well as fw values (*P* < 0.05). Nevertheless, there were no significant differences in Bin-DAI, Bin-ODI, ODI and Da values (*P* = 0.959, 0.709, 0.842, 0.543, respectively) between two groups (Fig. 3).

Table 2 showed the AUC values and its 95% confidence interval (95% CI), sensitivity as well as specificity values for discriminating whether patients with LrGG occurred recurrence within two years after resection. AFD-max, De^⊥^ and fw showed considerable diagnostic efficacy in prediction (AUC > 0.8) while AFD-sum, AFD-total, AWF, MD, RD, FA, AK, MK, RK, Bin-ICVF and ICVF showed acceptable diagnostic efficacy (AUC > 0.7). However, AD, De^∥^and f were limited in diagnosis (AUC < 0.7). De^⊥^ had the highest diagnostic efficacy and was significantly higher than those of De^∥^, f, RD, MK, Bin-ICVF and ICVF (*P* = 0.002, 0.009, 0.040, 0.040, 0.020, 0.020, respectively). The ROC results of Bin-DAI, Bin-ODI, ODI and Da were not applicable for the reason that these parameters showed no significant difference between two groups.

**Table 2:** ROC results of dMRI in discriminating recurrence after resection in LrGG

Parameters	AUC	95%CI	Sensitivity (%)	Specificity (%)
**AFD**				
AFD-max	0.805	0.679–0.931	82.6	72.0
AFD-sum	0.722	0.574–0.870	60.9	80.0
AFD-total	0.774	0.639–0.909	65.2	88.0
**AWF**				
AWF	0.703	0.550–0.856	65.2	76.0
**DTI**				
AD	0.682	0.528–0.836	69.6	68.0
MD	0.744	0.596–0.893	56.5	96.0
RD	0.762	0.615–0.908	65.2	88.0
FA	0.732	0.585–0.879	60.9	92.0
**DKI**				
AK	0.736	0.594–0.877	78.3	64.0
MK	0.767	0.631–0.903	82.6	86.0
RK	0.744	0.602–0.887	82.6	64.0
**Bingham NODDI**				
Bin-ICVF	0.751	0.606–0.896	69.6	76.0
**NODDI**				
ICVF	0.750	0.604–0.895	73.9	72.0
**SMI**				
De^∥^	0.624	0.465–0.783	95.7	36.0
De^⊥^	0.885	0.792–0.978	91.3	76.0
f	0.683	0.530–0.837	56.5	80.0
fw	0.830	0.713–0.947	82.6	76.0

**Fig. 4:**
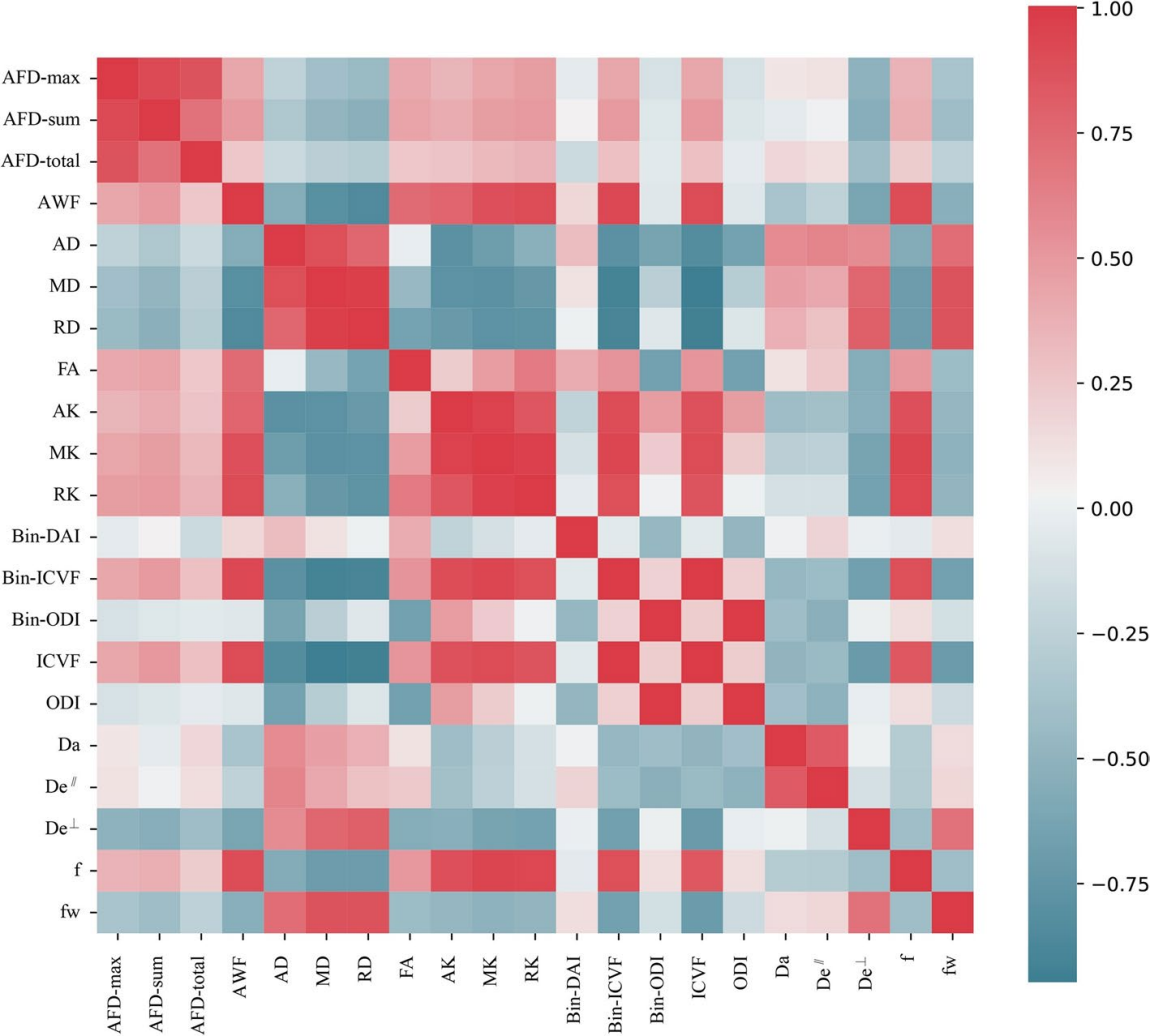
Correlation heatmap of various dMRI derived parameters. Red checks represented positive correlation while blue checks represented negative correlation. The more obvious the color was, the stronger correlation existed

**Fig. 5:**
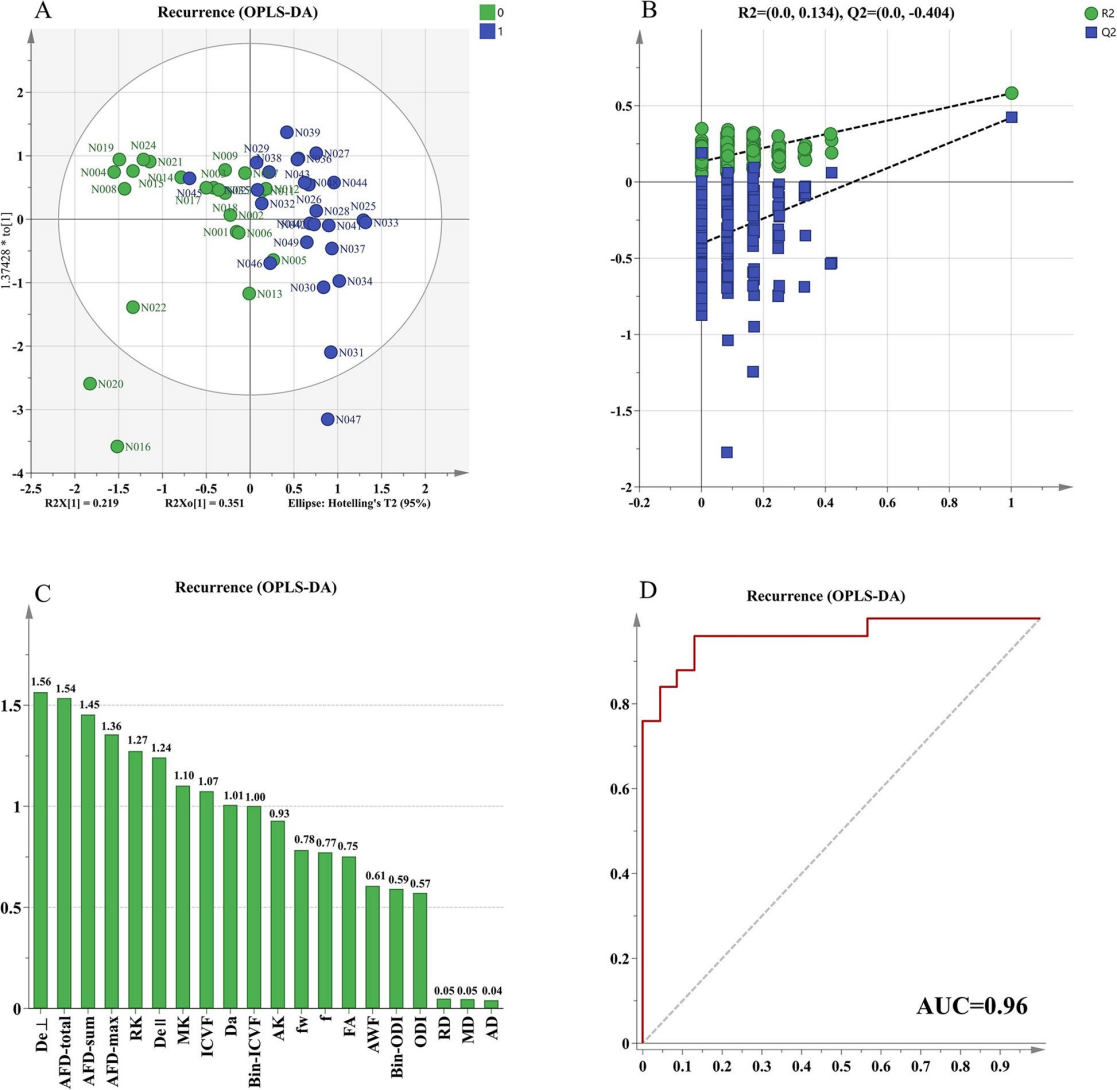
The results of OPLS-DA. **A** score scatter plot, green dots and blue dots are located on both sides of the y-axis respectively which means model reconstructed by OPLS-DA under comprehensive consideration of the correlation of various parameters can effectively distinguish two groups.; (**B**) 200 times permutation tests, this test aimed to prove whether model was overfitting. The results were consistently lower than the original R2Y and Q2Y values, with the intercepts on y-axis of R2Y and Q2Y under 0.3 and 0.05, respectively which suggested that the model fitted well without overfitting; (**C**) VIP plot, The variable importance for the projection(VIP) means the importance of parameters in identifying different groups, VIP value more than 1 indicates that the parameter is significant and the higher the VIP value, the greater the importance of the parameter in distinguishing between different groups. ; (**D**) ROC curve of multiparametric prediction model

There existed some relative correlation between dMRI derived parameters from different reconstruction models. For example, AWF and f had a strong positive correlation (Fig. 4). Figure 5 showed the results of OPLS-DA. Under comprehensive consideration of the correlation of various parameters, the multiparametric prediction model had great ability to diagnose postoperative recurrence in LrGG with its AUC value up to 0.96. The R2Y score of 0.581 and Q2Y score of 0.422 demonstrated acceptable model interpretability and predictability. Additionally, the results in 200 times permutation tests were consistently lower than the original R2Y and Q2Y values, with the intercepts of R2Y and Q2Y under 0.3 and 0.05, respectively. These suggested that the model fitted well without overfitting. Furthermore, several parameters were found to be valuable markers with their VIP values more than 1 on the premise that they showed significantly differences between groups (*P* < 0.05). The parameters in descending order of VIP values included De^⊥^, AFD-total, AFD-sum, AFD-max, RK, De^∥^, MK, ICVF and Bin-ICVF.

## Discussion

Our study discussed the application of advanced dMRI in predicting the postoperative recurrence of LrGG within two years after resection. The results showed that the lesion margin obtained from T2 FLAIR may helpful to assess the postoperative recurrence. AFD, AWF, DTI, DKI, Bingham NODDI, NODDI and SMI reconstruction models can provide valuable parameters for prediction. Among them, De^⊥^ showed the highest diagnostic efficiency and was significantly higher than others. In the further OPLS-DA, De^⊥^ also had the highest VIP value, and the multiparametric prediction model with comprehensive consideration of the correlation of various parameters had great diagnostic performance.

There have been no conclusive researches on the mechanism of glioma recurrence. By exome sequencing, Brett found that some gene mutations such as TP53 and ARTX were not detected in recurrent tumors compared to primary tumors in low grade gliomas [29]. Similarly, research focused on genomic analysis of primary and recurrent GBM indicated that there existed subclone alterations between them [30]. In addition, in the study of tumor microenvironment, researchers have found that there are differences in the immunological composition of extracellular tumor microenvironment between primary and recurrent GBM, with a decrease in invasive mononuclear cells and an increase in macrophages/microglia in recurrent GBM [31]. On the other hand, cancer stem cells (CSCs), as important roles, also participated in the process of tumor growth, metastasis and recurrence. Chen proposed that CSC would re-enter the cell cycle after treatment with temozolomide. Sequential administration of ganciclovir plus temozolomide to eliminate CSC could improve the survival of GBM mice, demonstrating the importance of CSC in mediating glioma recurrence after treatment [32]. These differences in transcriptome, genome, tumor microenvironment as well as the existence of CSCs suggested that recurrent gliomas had unique molecular biological characteristics. For patients with gliomas who may occur postoperative recurrence, the primary lesions may have specific imaging manifestations.

On T2 FLAIR imaging, differences were found in preoperative imaging between non-recurrence and recurrence groups. Specifically, lesions margin of non-recurrence group appeared sharper, while those of recurrence group appeared more blurred. Previous studies have shown that tumor cells of gliomas can migrate through white matter fiber bundles in the tumor parenchyma or peritumoral edema area, resulting in invasive growth or local metastasis and recurrence [33–35]. In this study, compared with cases in non-recurrence group, lesion margins of gliomas in recurrence group appeared more blurred for the reason that these lesions existed relatively severe peritumoral edema. And the characteristic of tumor aggressive growth was more obvious, which therefore leading them more likely to occur recurrence within two years after operation. The blurred boundary of gliomas is mainly caused by the following reasons: First, strong invasiveness of tumor cells: glioma cells can spread widely in normal brain tissue, so the boundary between tumor and normal brain tissue is not obvious. Then, the cellular heterogeneity within the glioma makes it difficult to distinguish part of the tumor area from the surrounding normal tissue. And more, the location and shape of the tumor are changeable, gliomas can occur anywhere in the brain and have a variety of forms, which further aggravates the difficulty of definition. The unclear tumor boundary has the following effects on the treatment and prognosis of glioma: surgical resection is difficult: surgery is one of the common methods for the treatment of glioma, but because of the unclear boundary, it is extremely difficult to remove the tumor completely. Residual tumor cells are an important cause of recurrence [36]. The difficulty of localization of radiotherapy and chemotherapy: treatment planning requires accurate tumor location to maximize efficacy and reduce damage to normal brain tissue. Unclear boundaries pose a challenge to treatment planning [37]. In addition, we found that the pathological grade of gliomas in non-recurrence group was higher than that in recurrence group. Postoperative recurrence of LrGG involves complex pathological processes that are not solely dependent on the initial pathological grade [1, 38]. Factors such as tumor microenvironment, CSCs, and genetic mutations play significant roles in recurrence. Research indicates that CSCs, which possess the ability to self-renew and drive tumor growth, can re-enter the cell cycle after treatment, contributing to tumor recurrence. Gliomas with a higher initial grade might have undergone more aggressive treatment, reducing the population of CSCs and thus lowering the recurrence rate. Additionally, recurrent gliomas often exhibit changes in their microenvironment, which might not be directly related to the initial pathological grade but rather to the dynamic interactions within the tumor and its surroundings. Moreover, gliomas are heterogeneous, and molecular characteristics can vary significantly even within the same pathological grade. Factors such as IDH1 mutation status, MGMT promoter methylation, and CDKN2A/B status can influence tumor behavior and recurrence risk independently of histological grade, which can affect recurrence patterns. Gliomas with a higher pathological grade might receive more aggressive treatment regimens, including extensive surgical resection, higher doses of radiotherapy, and more potent chemotherapeutic protocols. These treatments could effectively reduce the tumor burden and eliminate cells with high recurrence potential. In contrast, lower-grade gliomas might receive less aggressive treatment, leaving behind cells with a higher likelihood of recurrence due to incomplete eradication of invasive cells or CSCs.

According to previous studies, gliomas with postoperative recurrence potential had their own characteristics at genomic and transcriptome level, molecular and cellular level in tumor microenvironment. These characteristic manifestations were likely to result in macroscopic imaging features changes which were difficult to be reflected on conventional MRI structural imaging because of the low resolution. Advanced dMRI reflected the microscopic motion of water molecules, containing structural characteristic information of white matter fiber bundles, and could provide microstructure information which cannot be reflected by traditional MRI. dMRI has been widely used in clinical research and assessment of treatment response in gliomas [14, 16, 23, 39]. Therefore, this study assumed that gliomas with and without recurrence potential had different imaging manifestations on dMRI, and various reconstruction models had their own advantages. The establishment of a reasonable prediction model after taking fully account of the correlation between multiple models and their derived parameters will be helpful in predicting the postoperative recurrence of LrGG.

Based on fODF, AFD characterized the axonal density, which could analyze fiber bundle information within voxels [40]. The increase in AFD value in a sense reflected the increase of the number or volume of axons. AWF, derived from WMTI, was defined as the ratio of water inside the axon to the sum of water inside and outside the axon [41]. Similar to AFD, the increase in AWF value indicated an increase in axonal density and limited diffusion of water molecules. In this study, the preoperative AFD and AWF values of LrGG with postoperative recurrence were significantly higher than those of LrGG without postoperative recurrence. This finding might be due to the fact that LrGG with potential of recurrence exhibited more aggressive and malignant biological behavior. The accumulation of axons within the unit voxel in tumor parenchyma leaded to the reduction of extra-axonal space, and the complex structure of white matter fiber bundles restricted the dispersion of water molecules, resulting in an increase in AFD and AWF values.

Besides, for DTI and DKI, the preoperative MD value in recurrence group was significantly lower than that in non-recurrence group, while FA and MK values were higher. We suggested that the growth of glioma in recurrence group was more invasive and the tumor cells grown relatively faster, which resulted in the smaller extracellular space. Thus, the diffusion of water molecules in microenvironment became limited, which may explain the significantly lower MD value compared with non-recurrence group. FA represented the integrity and orientation of white matter fiber bundle, which had some correlation with cell proliferation and density [42, 43]. The more aggressive malignant growth pattern of gliomas in recurrence group propelled the increase in FA value. In a previous study, researchers found that for non-enhanced peritumoral region (NEPTR) of GBM, preoperative FA values in areas with postoperative recurrence were significantly lower than those in areas without recurrence [44]. NEPTR on T2 FLAIR mainly contained angiogenic edema and possible tumor cell infiltration. Previous researchers thought that there was a significant negative correlation between FA and tumor cell infiltration [43], which might due to the decrease of extracellular space and isotropic diffusion. Therefore, FA value of areas with recurrence potential in NEPTR would be significantly lower than those without recurrence potential, which was not inconsistent with the results of this study.

Previous research explore the use of DTI to identify patterns that predict glioma recurrence. It highlights the potential of DTI parameters like FA to delineate areas at risk for recurrence based on their diffusion properties, indicating that these techniques could provide valuable insights into the structural and pathological nature of gliomas [39]. Another research imaged 26 patients with gliomas using DTI. Patients were imaged after 2 years or on symptomatic tumor recurrence. The diffusion tensor was split into its isotropic (p) and anisotropic (q) components, and these were plotted on T(2)-weighted images to show the pattern of DTI abnormality. This was compared to the pattern of recurrence. Diffusion tensor imaging is able to predict patterns of tumor recurrence [45]. And more, researchers evaluate diffusion-proliferation models informed by structural MRI and DTI, after tumor resection. They applied the models to a unique longitudinal dataset of 14 patients with LGG, who received no treatment after surgical resection, to predict the recurrent tumor shape after tumor resection. The DTI-informed model reached the best predictive performance. There is a significant improvement in the prediction of the recurrent tumor shape when using a DTI-informed anisotropic diffusion model with respect to istropic diffusion [46]. Like our study, they found high diagnostic efficacy of these imaging markers in assessing tumor recurrence potential, multiple dMRI metrics including DTI were employed to distinguish between recurrent and non-recurrent gliomas. These findings align with our research, underscoring the effectiveness of sophisticated MRI techniques, including DTI and other dMRI metrics, in predicting glioma recurrence. This validation from multiple studies highlights the relevance of advanced imaging techniques in improving the prognosis assessment and potentially guiding treatment strategies for glioma patients.

In addition, for Bingham NODDI and NODDI, values of Bin-ICVF and ICVF in recurrence group were higher. ICVF was defined as intracellular volume fraction, which represented the density of axons. The higher the density of glioma cells, the higher the ICVF value in recurrence group. Bin-DAI and ODI, which indicated the degree of water dispersion limitation didn’t show significant difference between groups. This might be due to the fact that the establishment of NODDI model was not sensitive to the prediction of postoperative recurrence of LrGG, which needed further confirm. For SMI, the recurrence group had higher De^∥^ and f values and lower De^⊥^ and fw values, while the difference in Da between groups was not statistically significant. The more malignant growth pattern of gliomas in recurrence group made the destruction of white matter fiber bundles more serious and the tumor microenvironment more complex, which might be the reason for the increase of axonal signal fraction, f. The reduced De^⊥^ value indicated the decrease of the extra-axonal space, and the dispersion of water molecules in the vertical axon direction was relatively limited, which was probably caused by the increase of tumor cell density in recurrence group. However, De^∥^ ascended in recurrence group, which meant that the dispersion of water molecules in the parallel axon direction was relatively unrestricted.

Moreover, in order to explore the predictive ability of various derived parameters of dMRI for postoperative recurrence of LrGG, the results of ROC analysis showed that except for the limited diagnostic efficiency of AD and De^∥^ (AUC < 0.7), other parameters had great diagnostic efficacy, among which AFD-max, De^⊥^ and fw had considerable diagnostic efficiency (AUC > 0.8). De^⊥^ performed best (AUC = 0.885), which was significantly higher than others. Although a single parameter alone showed great diagnostic efficiency, the reconstruction models and their derived parameters were obtained from the same original dMRI data, which leaded to some correlation among parameters. For example, there existed a strong positive correlation between AWF representing axon water fraction and f representing axonal signal fraction. It is necessary to comprehensively consider the correlation among the parameters in order to improve the diagnostic efficiency and reduce the diagnostic error. Therefore, in this study, a multiparametric prediction model was established by using OPLS-DA method. Compared with a single parameter alone, its prediction efficiency for postoperative recurrence of LrGG was improved to 0.96. The variable importance for the projection (VIP) value of De^⊥^ was the highest. Regardless of the predictive ability of a single derived parameter or the evaluation of VIP in multiparametric prediction model, De^⊥^ exhibited the best performance, suggesting that De^⊥^ was likely to become a valuable imaging marker for predicting postoperative recurrence of LrGG.

This study still had some limitations. First of all, this study only conducted in one medical institution, the number of cases was relatively small, and the adoption of retrospective research lacked prospective validation. In future, data from multiple centers and large samples will be needed to verify the effectiveness of dMRI in predicting postoperative recurrence of gliomas. In addition, the molecular biological characteristics such as IDH1 genotype and MGMT promoter methylation status had not been subdivided in analysis and these genotypic or epigenetic differences might also affect postoperative recurrence. This study will continue to analyze and explore different subgroups of molecular biological characteristics of LrGG.

AFD, AWF, DTI, DKI, Bingham NODDI, NODDI and SMI models of advanced dMRI had great performance in evaluating the recurrence of LrGG within two years after operation. The derived parameter of SMI model, De^⊥^, had the best diagnostic effectiveness and was significantly better than others. The information such as lesion margin provided by traditional MRI was also helpful to predict postoperative recurrence. The combination of multiparametric advanced dMRI information will make convenient for diagnosing as well as making clinical decisions, such as appropriate expansion of the resection scope and preventive use of anti-angiogenic drugs and so on, which had certain clinical significance.

## Data Availability

The datasets used and/or analyzed during the current study are available from the corresponding author on reasonable request.

## Abbreviations

IDH1: isocitrate dehydrogenase 1
LrGG: lower-grade glioma
DTI: diffusion tensor imaging
DKI: diffusion kurtosis imaging
WMTI: white matter tract integrity
NODDI: neurite orientation dispersion and density imaging
AFD: apparent fiber density
SMI: standard model imaging
FA: fractional anisotropy
MD: mean diffusivity
AD: axial diffusivity
RD: radial diffusivity
MK: mean kurtosis
AK: axial kurtosis
RK: radial kurtosis
ICVF: intra-cellular volume fraction
AWF: axonal water fraction
OPLS-DA: Orthogonal Partial Least Squares-Discriminant Analysis

## Data availability

No datasets were generated or analysed during the current study.
